# Retraction Notice

**DOI:** 10.5713/ajas.2003.77RT

**Published:** 2020-04-20

Ethical committee of Asian-Australasian Journal of Animal Sciences (AJAS) reviewed possible duplicate publication of the following two articles and found that two articles are essentially identical. According to AJAS Ethics Guideline, the article (AJAS Vol 16 No 1 pp 77–82) will be retracted.

**Articles involved:**

1. Effect of Dietary Formic and Propionic Acids Mixture on Limiting Salmonella pullorum in Layer Chicks, Y. H. Al-Tarazi and K. Alshawabkeh, AJAS Vol16 No1 P77–82 (Published on 1 January, 2003).
2. Effect of Dietary Formic and Propionic Acids on Salmonella Pullorum Shedding and Mortality in Layer Chicks after Experimental Infection, Y. H. Al-Tarazi and K. Alshawabkeh, J. Vet. Med. B 50, 112–117 (Published on 21 March 2003).

